# Efficacy of *Aspergillus tubingensis* GX3′ Fermentation against *Meloidogyne enterolobii* in Tomato (*Solanum lycopersicum* L.)

**DOI:** 10.3390/plants12142724

**Published:** 2023-07-21

**Authors:** Aatika Sikandar, Fukun Gao, Yixue Mo, Qian Chen, Rana Muhammad Kaleem Ullah, Haiyan Wu

**Affiliations:** State Key Laboratory for Conservation and Utilization of Subtropical Agro-bioresources, Guangxi Key Laboratory of Agric-Environment and Agric-Products Safety, College of Agriculture, Guangxi University, Nanning 530004, China; aatika_sikander@yahoo.com (A.S.); 18854881887@163.com (F.G.); mo-yixue@st.gxu.edu.cn (Y.M.); cqsdau@163.com (Q.C.); ranakaleem193@gmail.com (R.M.K.U.)

**Keywords:** tomato, root-knot nematodes, nematicides, biocontrol, biomass, fermentation

## Abstract

*Meloidogyne enterolobii* is one of the most virulent root-knot nematodes (RKNs). *Aspergillus tubingensis* Raoul Mosseray, 1934, is used to produce bioactive substances, enzymes, and secondary metabolites. However, no research has been conducted yet on the efficacy of *A. tubingensis* against plant-parasitic nematodes. Thus, the novel research was planned to evaluate the biocontrol efficacy of *A. tubingensis* fermentation against *M. enterolobii*. The findings showed that egg hatching inhibition and mortality of *M. enterolobii* increased with increasing concentration of fermentation and exposure time. The maximum second-stage juveniles (J2s) mortality was achieved via 100% fermentation at 72 h. Similarly, 100% fermentation inhibited 99.9% of egg hatching at 8 d. *A. tubingensis* fermentation increased plant biomass, decreased second-stage juvenile invasion, and inhibited nematode development and reproduction in greenhouse conditions. *A. tubingensis* reduced J2 invasion into tomato roots by 42.84% with CS+ (coated seeds plants with nematodes inoculum) and 27.04% with T+ (100% fermentation broth and nematodes inoculum both) treatments. Moreover, CS+ and T+ treatments decreased nematode development by 54.31% and 21.48%, respectively. It is concluded that the *A. tubingensis* GX3 strain can be used as a novel microbial biocontrol agent against *M. enterolobii*.

## 1. Introduction

Nematodes are among the most common creatures in the world [1]. Plant-parasitic nematodes (PPNs) cause a substantial risk to agriculture. It is estimated that they are responsible for a loss of more than USD 173 billion per year in agricultural output throughout the globe [2]. Root-knot nematodes (RKNs) are recognized as one of the most destructive pests of vegetables, and their economic impact is tremendous [3,4]. They are obligate endoparasites that may be found in the roots of over 3000 diverse species of plants [5]. Moreover, their abundance increases when environmental conditions are ideal [6].

*Meloidogyne enterolobii* Yang and Eisenback, 1983 (guava root-knot nematode), constitutes a threat to agriculture owing to the fact that it may be distributed throughout the world and infect a wide range of hosts [7]. This species is one of the most virulent RKN species owing to its capacity to emerge and reproduce on resistant cultivars of the most prevalent tropical RKN species [8]. *M. enterolobii* may be responsible for more than 65% of the agricultural losses, much greater than any other RKN species [9]. The effects of *M. enterolobii* may include a decline in yield quality and quantity [10]. Below-ground symptoms, such as root galls and numerous above-ground signs, include wilting, leaf yellowing, and stunted development [11]. After infection with *M. enterolobii*, plants are more susceptible to secondary plant infections, such as *Fusarium solani* (von Martius, 1842) parasitizing guava [12]. *M. enterolobii* management is complex because of its diverse host range and rapid reproduction cycles [9]. Therefore, developing successful strategies and incorporating them into disease management programs might effectively prevent disease outbreaks, lower disease severity, and boost agricultural output [13].

The tomato (*Solanum lycopersicum* L.), a widely grown plant in the Solanaceae family, is a globally significant vegetable [14]. China is the world’s top tomato producer [15]. However, RKNs pose a major threat to tomato production, resulting in yearly losses of up to 70 billion US dollars worldwide [16]. Thus, strategies for preventing and managing RKN disease are urgently needed. While the world’s rising population has a need for food, the agriculture and forestry industries are presently facing serious problems from phytopathogens such as PPNs. At the same time, nematicides have made a significant contribution to agricultural output over the last half-century. Unfortunately, the inappropriate use of chemical agents caused adverse side effects on environmental and agricultural sustainability [17]. Methyl bromide, dibromochloropropane, ethylene dibromide (EDB), and dibromochloropropane are some of the nematicide chemicals taken off the market due to their propensity to cause severe diseases [18].

Biocontrol is a better approach than chemical control regarding both safety and environmental friendliness [19]. The antagonists and nematophagous microbes exhibit tremendous potential as prospective substitutes for nematicides [20]. China has one of the world’s fastest economic growth rates but has serious environmental issues [21]. Since a decade ago, China has paid greater attention to pollution control, environmental preservation, and ecological restoration [22]. The microbial biocontrol agent industry in China has undergone a golden era of tremendous expansion [23]. Meanwhile, several issues still impede the industry’s further expansion in China. Only a few nematophagous bacteria and fungi are currently commercially available worldwide for use in eradicating plant parasitic nematodes [24].

Fungal biocontrol agents use diverse approaches for capturing nematodes through sticky branches, constricting and non-constricting rings, killing them through the release of harmful substances, and digesting them through the colonization of their reproductive systems [25]. *Acremonium*, *Alternaria*, *Arthrobotrys*, *Aspergillus*, *Chaetomium*, *Cladosporium*, *Clonostachys*, *Diaporthe*, *Drechslerella*, *Epiccocum*, *Fusarium*, *Gibellulopsis*, *Melanconium*, *Metacordyceps*, *Monacrosporium*, *Neotyphodium*, *Paecilomyces*, *Penicillium*, *Phialemonium*, *Phyllosticta*, *Piriformospora*, *Purpureocillium*, *Talaromyces*, and *Trichoderma* are antagonistic to nematodes via the production of secondary metabolites [26,27,28]. The saprophytic fungi decrease root-knot nematode penetration in roots [25]. Obligatory arbuscular mycorrhizal fungi potentially alter the host-finding behavior of *Meloidogyne* species [29]. However, fundamental research on microbial action mechanisms is still lacking, and novel agents for simple applications should be developed.

*Aspergillus* spp. are among the oldest fungi species identified, ubiquitous in diverse environments, and can survive harsh environmental conditions [30]. Black aspergilli are notable due to their rapid growth, enormous abundance, pH tolerance [31], and ability to produce hazardous metabolites such as malformins, napthopyrones, and ochratoxin [32]. In addition, *Aspergillus* species also produce a diverse array of secondary metabolites [33]. The production of these compounds by *Aspergillus* species enables their use as microbial biocontrol agents against plant-parasitic nematodes [34]. Several secondary metabolites were identified from several *Aspergillus* species, including alkaloids, butenolides, and cytochalasins, which have varied biological actions, including anti-inflammatory, antibacterial, and antimicrobial [35]. *A. niger* F22 generates oxalic acid (OA), which is used to prevent root-knot nematodes [36]. According to Siddiqui et al. [37], six species of *Aspergillus* exhibited highly efficient predation and nematicidal action against *M. javanica* species and produced a diverse array of secondary metabolites. *A. tubingensis* is used to produce bioactive substances, enzymes (such as acid phosphatase, glucose oxidase, xylanase, phytase, lipase, amylase, and xylosidase), and particular metabolites such as asperazine in industrial fermentation [38]. *A. tubingensis* has become particularly important in agriculture because it produces glucose oxidases and other metabolites that may be utilized as biofungicides, which protect plants from fungal infections [39]. Soil amendment with *A. tubingensis* has been demonstrated to be capable of improving maize yield by dissolving phosphates in soil and decreasing alkalinity in bauxite wastes [40]. *A. tubingensis* is more likely to survive in these applications because of its resilience to high pH environments [40]. It has been reported that *A. tubingensis* acts as a microbial biocontrol agent to protect tomato plants against the pathogenic fungus *Fusarium solani* [41]. However, no research has yet to be conducted on the efficacy of *A. tubingensis* against *M. enterolobii*.

Thus, the current study aimed to evaluate the effectiveness of *A. tubingensis* GX3′ fermentation. The efficiency of the GX3 strain against *M. enterolobii* was confirmed under laboratory and greenhouse conditions. The present study also planned to explore the nematicidal activity of *A. tubingensis* GX3 fermentation to control *M. enterolobii* by seed coating on tomato seeds. This study provides a theoretical basis for creating a commercially feasible and beneficial microbial biocontrol agent.

## 2. Results

### 2.1. Effect of Fermentation Filtrate on M. enterolobii Juveniles (J2s)

**Figure 1:**
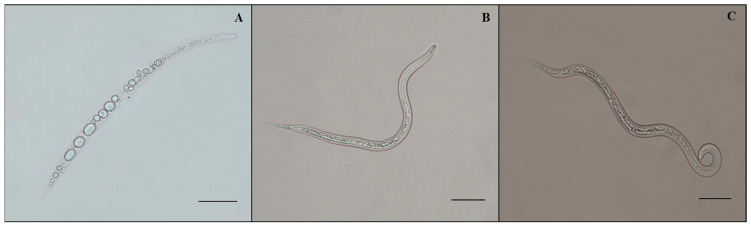
Morphology of *M. enterolobii* juveniles treated with *A. tubingensis* GX3 fermentation. (**A**) J2s in 100% fermentation at 24 h; (**B**) J2s in sterilized water at 24 h; (**C**) J2s in 20% Czapek medium at 24 h. Bar: (**A**–**C**) = 100 μm.

The data in Table 1 represent that the average percent mortality in the fermentation of *A. tubingensis* was significantly different from that in the Czapek medium. Therefore, it is stated that the medium had no nematicidal or nematostatic effect because the mean mortality observed in the medium was statistically similar to that in distilled water. All concentrations of fermentation had a nematicidal effect on *M. enterolobii*. The maximum J2s mortality was recorded at a concentration of 100% fermentation, which reached 100% (*p* < 0.0001) after 72 h. J2s mortality was 22.6%, 47.2%, 78.8%, 99%, and 100% after 6, 12, 24, 48, and 72 h with 100% concentration of *A. tubingensis* fermentation. Increases in fermentation concentrations resulted in greater J2 mortality. Second-stage juveniles progressively decreased their mobility after 24 h and were immobile by 72 h. After being exposed to *A. tubingensis* fermentation, the immobile second-stage juveniles were put into distilled water for 12 h, but they exhibited no recurrence of motility. At the same time, microscopic examination indicated that juveniles became inactive, bradykinetic, and paralyzed when J2s were exposed to *A. tubingensis* fermentation. At 72 h, the cuticle was crimped, and the intestine was removed. As the exposure time increased, the gut and esophagus intersection area was progressively damaged. Furthermore, the rigid nematode bodies had an unclear contour of anterior structures, creating vacuoles and constrictions (Figure 1A). At the same time, the J2s in distilled water and Czapek medium showed normal activity, and no morphological abnormalities were noticed (Figure 1B,C).

### 2.2. Effect of Fermentation Filtrate on Meloidogyne enterolobii Eggs Hatching

**Figure 2:**
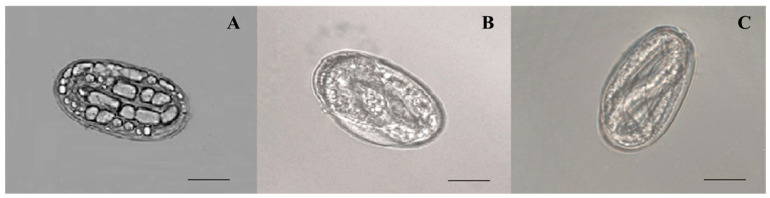
Morphology of *M. enterolobii* eggs treated with *A. tubingensis* GX3 fermentation. (**A**) eggs in 100% fermentation at 4 d; (**B**) eggs in 20% Czapek medium at 4 d; (**C**) eggs in sterilized water at 4 d. Bar: (**A**–**C**) = 100 μm.

The nematicidal potential of *A. tubingensis* against *M. enterolobii* was evaluated based on its ability to suppress egg hatching (Table 2). Egg masses that had reached maturity were chosen, and it was ensured that all of the eggs were at the same embryonic stage. The mean hatching rates in the Czapek medium were statistically equal to those in distilled water, indicating that the medium exhibited neither an ovicidal nor an ovistatic feature. The hatching of eggs was considerably stymied by *A. tubingensis* fermentation filtrate at various concentrations, with the inhibitory impact becoming more prominent as the filtrate concentration increased. A total of 0.1%, 0.2%, 0.6%, 5.4%, 16%, and 26.6% of eggs hatched 8 days (*p* < 0.0001, F = 710.987) after incubation in 100%, 75%, 50%, 25%, 10%, and 5% fermentation broth, respectively, which is considerably lower than the control groups (distilled water, 92%; 20% Czapek medium, 87.2%). The fermentation of *A. tubingensis* had the greatest effect on preventing hatching eggs at 100% concentration. The ability to prevent *M. enterolobii* eggs from hatching was shown to rise in direct proportion to both the concentration and duration of exposure. The viability of eggs that were still unhatched was determined by putting them in distilled water; however, they did not hatch after being in the water for the required time. The microscopy observation indicated that an egg’s contents disintegrated after fermentation exposure. Therefore, the dense vacuoles that developed in eggs made them unable to hatch (Figure 2A). In contrast, the eggs in distilled water and Cazpek medium seemed to develop properly and normally hatch (Figure 2B,C). It was determined that the *A. tubingensis* fermentation filtrate had a high potential for ovicidal activity by causing damage to the egg structure.

### 2.3. Hatching Inhibition

**Figure 3:**
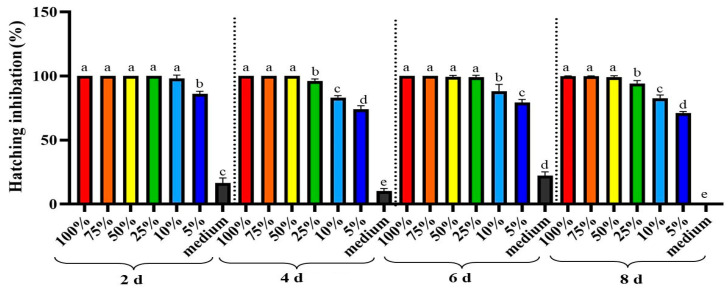
Hatching inhibition rate of *M. enterolobii* eggs after different days of exposure with *A. tubingensis* GX3 fermentation. Data represent the Mean ± Standard error. The different letters on bars are significantly different according to Duncan’s multiple range test (*p* > 0.05).

After incubating *M. enterolobii* eggs in *A. tubingensis* GX3 fermentation for 8 days, the egg hatching rate was reduced relative to that of the untreated control (Figure 3). Based on the experiment results, all concentrations of *A. tubingensis* GX3 fermentation significantly (*p* < 0.0001) inhibited the hatching of eggs after 2, 4, 6, and 8 d. The complete inhibitory effect was observed in 100%, 75%, and 50% concentrations of fermentation because no egg was hatched after exposure to these concentrations. In contrast, Czapek medium had no significant inhibitory effect on egg hatching.

### 2.4. Probit Analysis of Ovicidal and Larvicidal Assays

The outcomes of the probit analysis showed the LC_50_ values, the Chi-square, and the confidence limits at the 95% confidence interval (Table 3). In contrast, the lowest LC_50_ value of 13.09% was reported in the larvicidal test during a 72 h exposure period, followed by values of 39.71%, 59.22%, 141.39%, and 437.76% at 48, 24, 12, and 6 h exposure periods, respectively. Similarly, the lowest LC90 value was 34.893 after an exposure duration of 72 h, followed by 84.40%, 413.67%, 1693.06%, and 4357.15% after exposure periods of 48, 24, 12, and 6 h, respectively. The LC_50_ and LC_90_ values both decreased as the exposure time was prolonged. The filtrate of the *A. tubingensis* fermentation showed considerable toxic activity against *M. enterolobii* juveniles, as evaluated through an in vitro assay.

The data presented in Table 4 show the median effect concentrations (EC_50_ and EC_90_) of *A. tubingensis*’ fermentation on hatching at different days of exposure. During the ovicidal test, the EC_50_ value that was found to be the lowest was 2.36% after being exposed for 2 days, followed by 2.32%, 2.92%, and 3.55% after being exposed for 6, 4, and 8 days, respectively. In a similar manner, the lowest EC90 was 5.60% at 2 days, followed by 10.40%, 12.05%, and 13.74% at 6, 4, and 8 days, respectively.

### 2.5. Model Validation of Ovicidal and Larvicidal Assays

The results of the model validation on mortality and egg hatching in response to *A. tubingensis* fermentation treatment at various concentrations and time intervals are given in Table 5 and Table 6, respectively. The results of the concentration of the fermentation showed that the R2 values of the larvicidal at 100%, 75%, 50%, 25%, 10%, 5%, medium, and CK were 0.93, 0.98, 0.94, 0.83, 0.92, 0.95, 0.85, and 0.89, correspondingly. These values indicated improved performance compared to the RMSE (0.988–10.08). However, in the ovicidal assay, the concentrations of fermentation revealed that R2 values were 0.6, 0.6, 0.9, 0.95, 0.95, 0.99, 0.99, and 0.93 at 100%, 75%, 50%, 25%, 10%, 5%, medium, and distilled water (control), respectively, along with RMSE values ranging from 0.024 to 6.315, showing the most excellent curvature and best fitness of the model to the calculated data (Table 5).

Table 6 shows the model validation results for the effects of exposure duration on larvicidal and ovicidal assays. Time-dependent analysis of the larvicidal test showed that its performance improved with increasing exposure time, with *R*^2^ values of 0.87, 0.95, 0.9, 0.92, and 0.92 at 6, 12, 24, 48, and 72 h, respectively, outperforming the relative mean standard error (2.756–10.65). However, the *R*^2^ values of ovicidal at 2, 4, 6, and 8 days of exposure were 0.66, 0.76, 0.73, and 0.77, respectively, representing the best curve and best fit to the obtained data.

### 2.6. Greenhouse Experiment

#### 2.6.1. Seed Germination

**Figure 4:**
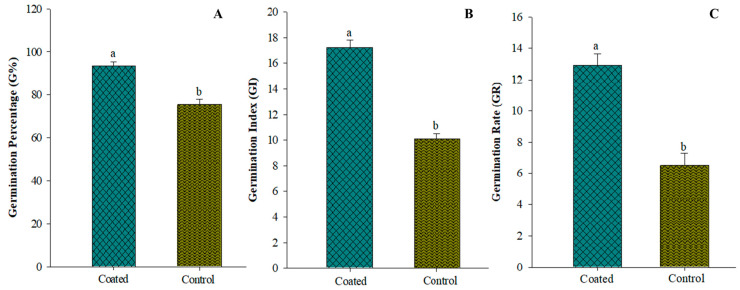
(**A**) Effect on germination percentage of tomato after seeds coated with GX3 fermentation (**B**) Effect on germination index of tomato after seeds coated with GX3 fermentation (**C**) Effect on germination rate of tomato after seeds coated with GX3 fermentation. Data represent the Mean ± Standard error. Different letters on bar indicate that values are significantly different according to Duncan’s multiple range test at *p >* 0.05.

Figure 4A–C depict the impact of fermentation on seed germination. It was noted that there were significant differences between the treatments. Germination percentages were significantly improved by the seed coating applied to the seeds. Coating seeds with *A. tubingensis* fermentation broth effectively increased germination percentage (G %) by 23.8% compared to the control. The seed germination index (GI) displayed the potency *A. tubingensis* fermentation by increasing to 69.96%. Similarly, the seeds coated with GX3 fermentation showed a 98% higher germination rate than the control group.

#### 2.6.2. Growth Parameters

**Figure 5:**
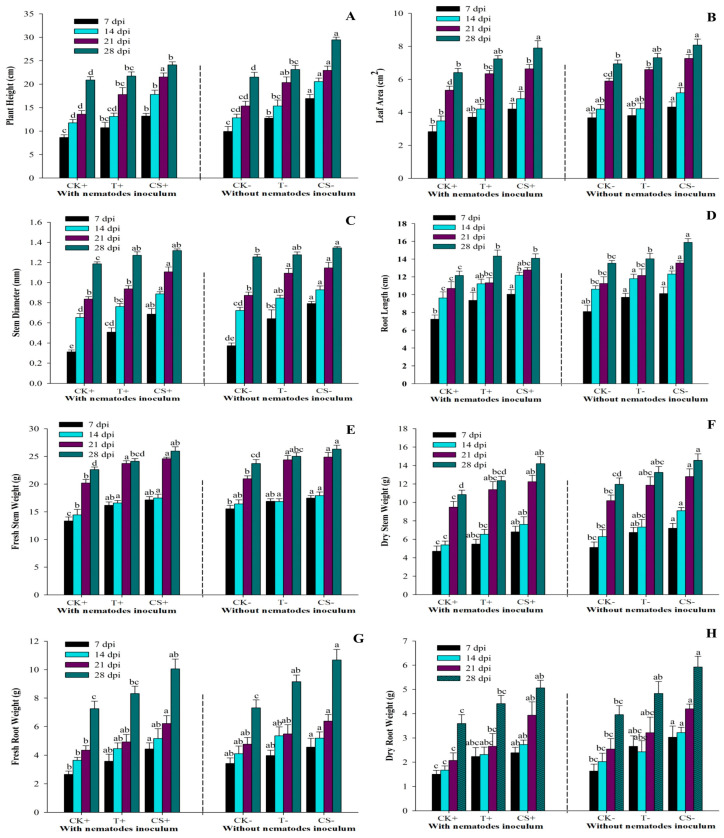
Effect of *A. tubingensis* fermentation on growth parameters of tomato plants with and without infection with *M. enterolobii* (**A**) Plant height, (**B**) Stem diameter, (**C**) Leaf area, (**D**) Root length, (**E**,**F**) Fresh and dry stem weight, (**G**,**H**) Fresh and dry root weight. Data represent the Mean ± Standard error of growth parameters. The different letters on bars are significantly different according to Duncan’s multiple range test (*p* > 0.05), whereas CK+ (control with nematodes inoculum); T+ (100% fermentation broth and nematodes inoculum both); CS+ (coated seeds plants with nematodes inoculum); CK− (control without nematodes inoculum); T− (100% fermentation broth only); CS− (coated seeds plants without nematodes inoculum).

Figure 5 demonstrates that all treatments effectively stimulated plant growth. The maximum plant height in the CS− treatment was 16.94, 20.56, 22.94, and 29.46 cm at 7, 14, 21, and 28 dpi, while the minimum plant height in the CK+ treatment was 8.64, 11.76, 13.6, and 20.9 cm at 7, 14, 21, and 28 dpi, respectively. At 7, 14, 21, and 28 dpi, the CS− treatment had significantly (*p* < 0.05) larger stem diameters: 0.792, 0.929, 1.146, and 1.345 mm, respectively. At 7, 14, 21, and 28 dpi, the leaf areas ranged between 2.83–4.32 cm^2^, 3.48–5.18 cm^2^, 5.34–7.26 cm^2^, and 6.41–8.07 cm^2^, respectively. The maximum fresh and dried stem weights recorded in CS− treatment at 28 dpi were 26.33 and 14.57 g, respectively. Similarly, the largest fresh and dried root weights were observed in CS− treatment at 28 dpi (10.68 and 5.92 g), whereas the lowest weights were recorded in CK+ at 7 dpi (2.6 and 1.5 g). Moreover, at 7, 14, 21, and 28 dpi, the greatest root length was 10.12, 12.32, 13.56, and 15.88 cm, respectively, for CS− treatment. The minimum root length was 7.24, 9.62, 10.7, and 12.16 cm in the CK+ treatment at 7, 14, 21, and 28 dpi, respectively. The pathogenicity of root-knot nematodes (RKN) decreased plant growth considerably (*p* < 0.05), while *A. tubingensis* fermentation filtrate-treated plants had significantly greater biomass than the control plants. The findings show that seed coating with *A. tubingensis* fermentation promotes tomato plant growth more effectively than other treatments.

#### 2.6.3. Nematode Infection

**Figure 6:**
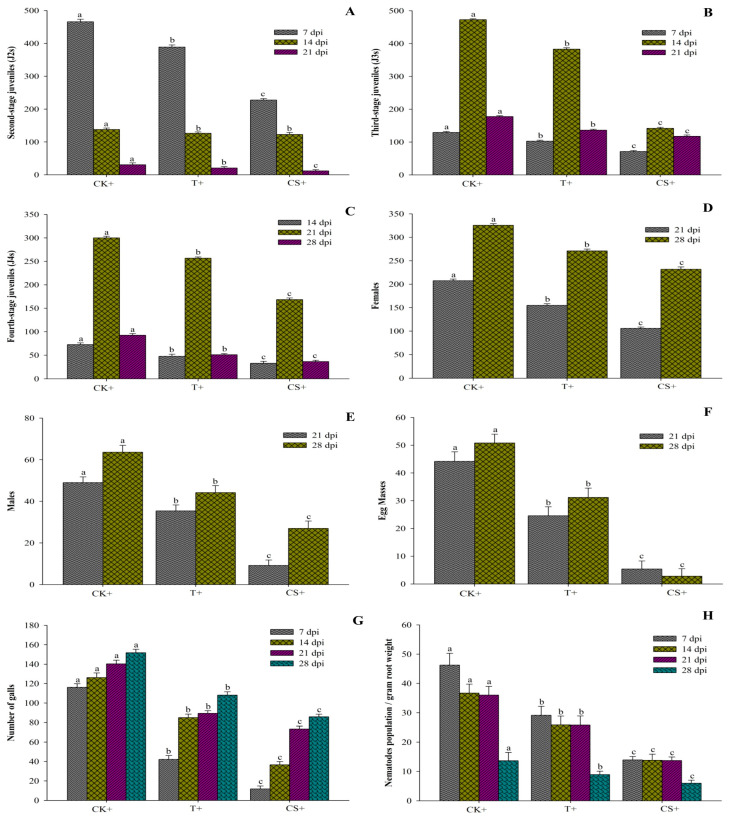
Effect of *A. tubingensis* fermentation against *M. enterolobii* in tomato. (**A**) Number of second-stage juveniles (J2s) in root system, (**B**) Number of third-stage juveniles (J3s) in root system, (**C**) Number of fourth-stage juveniles (J4s) in root system, (**D**) Number of females in root system, (**E**) Number of males in root system, (**F**) Number of egg masses on root system, (**G**) Number of galls per root system, (**H**) Number of nematodes present in per gram of root. Data represent the Mean ± Standard error. The different letters on bars are significantly different according to Duncan’s multiple range test (*p* > 0.05), whereas CK+ (control with nematodes inoculum); 2 = T+ (100% fermentation broth and nematodes inoculum both); 3 = CS+ (coated seeds plants with nematodes inoculum).

The impact of *A. tubingensis* on *M. enterolobii* populations was evaluated under greenhouse conditions using fermentation broth and seed coating. The plants were inspected regularly to examine the various stages of nematodes in the roots at 7, 14, 21, and 28 days (days post inoculums). The data in Figure 6 show that *A. tubingensis* effectively decreased the penetration rate of J2 into tomato roots, such that CS+ treatment reduced invasion by 42.84%, followed by T+ treatment by 27.04% compared to the control. It also suppressed the development of nematodes (J3 and J4), with CS+ treatment lowered by 54.31% and T+ treatment reduced by 21.48% compared to the control. Similarly, the rate of reproduction in T+ and CS+ treatments was lowered by 21.71% and 42.12%, respectively, compared to the control. It was also discovered that treated plants produced fewer females and males, forming fewer egg masses than the controls. When the fermentation broth was administered to inoculated nematodes in pots, the egg masses decreased by 41.26%, while it decreased by 91.37% when the seeds were coated. Similarly, when the fermentation broth was administered to nematode-inoculated pots, the number of galls and number of nematodes per gram of root weight dropped by 39.24% and 26.83%, respectively, as compared to the control. However, in seed-coated plants, the number of galls and nematodes per gram of root weight significantly declined by 61.17% and 61.27%, respectively, compared to control. The control plants exhibited a maximum population of nematodes at all DPIs, while the seed treated with *A. tubingensis* had much fewer nematodes.

## 3. Discussion

Nematode control using antagonistic microbes is a suitable method; nevertheless, more research is still necessary [20]. *Aspergillus* species are abundant in agricultural and non-agricultural soils, and they are known to be toxic to a wide range of plant-parasitic nematodes [42]. *A. tubingensis* is a promising alternative option for metabolite synthesis [38]. However, no study has been conducted about the use of *A. tubingensis* to suppress *M. enterolobii* in tomato. As a result, the current study was carried out to investigate the fermentation potential of *A. tubingensis* against *M. enterolobii* and its impact on plant growth. Overall, the outcome demonstrated that *A. tubingensis* GX3 exhibited strong nematicidal potential.

The current investigation provided evidence that *A. tubingensis* considerably increases the mortality rate of juvenile *M. enterolobii* and reduces egg hatching. It was discovered that the mortality and egg hatching rates in the Czapek medium were similar to those observed in the distilled water. Our results corroborated Sharapova [43] and Wu et al. [44], who reported that the medium exhibited no nematicidal ability. Our findings also showed that all fermentation concentrations had a nematicidal impact on *M. enterolobii*. J2 mortality reached 100% after 72 h with 100% and 75% concentrations of fermentation. The mortality response depends on the concentration and duration of exposure. Our findings are similar to those of Wang et al. [45] and Fan et al. [46], who found a clear correlation between the concentration of the fermentation and the duration of exposure time. According to the findings of previous studies, *A. niger* culture filtrate inhibited the rate of J2 viability and egg hatching of *Meloidogyne* species [36,47]. Moreover, more than half of *M. incognita* juveniles died after exposure to a 25% concentration of *Aspergillus* sp., *Fusarium* sp., *Penicillium* sp., and *Trichoderma* sp. fungal culture filtrate after 24 h of exposure [48]. Similarly, Hu et al. [49] reported that the mortality of juveniles depends upon *Chaetomium globosum* (Tveit and Moore, 1954) NK102 filtrate concentrations and exposure time. Moreover, the concentration of *Beauveria bassiana* (Hamill et al., 1969), *Metarhizium anisopliae* (Metschnikoff, 1879), and *Paecilomyces lilacinus* (Bainier, 1907) culture filtrates were directly related to hatching inhibition [50]. *Penicillium chrysogenum* (Thom, 1910) fermentation can efficiently parasitize juveniles inside the eggs and significantly restrict the number of eggs hatching [3]. Moreover, in the natural environment, the fungus conidia and hyphae readily enter the eggs, which ultimately leads to the death of the larvae inside the egg in the soil [51]. Similarly, *P. lilacinus* and *P. variotii* produced proteases and chitinases to break down nematode eggshells, caused extensive vacuoles in the chitin layer, exhibited a split in the vitelline layer, and lost its integrity [52]. *Trichoderma* secretes hydrolytic enzymes such as trypsin-like protease PRA1 [53], serine protease SprT [54], and chitinolytic enzymes chi18-5 and chi18-12 [55]. These extracellular hydrolytic enzymes allow the fungus to parasitize nematode eggs and juveniles. Similarly, *Aspergillus* species can generate hydrolytic enzymes, organic acids, and low-molecular-weight natural products that perform a variety of tasks, including P solubilization, as well as calcium and iron phosphate solubilization [56,57]. As a result, they have enormous potential for the creation of biofertilizers, which contribute to soil fertility and promote plant growth, both of which are critical in sustainable agriculture [58], as well as exhibit the biocontrol potential against various nematodes by producing various enzymes [36,47]. In the present study, we speculate that *A. tubingensis*’ fermentation secretes some form of enzymes or nematicidal material that destroys eggs and J2s, halting the organism’s development and finally causing its demise.

The application of fungi (such as *Trichoderma* spp., *Penicillium* spp., etc.) to seeds increased the germination percentage, germination index, and germination rate. In previous studies, seed coating with fungus fermentation improved seed germination and seedling vitality and decreased the incidence of pathogenic infections [59]. Sikandar et al. [60] reported that cucumber (*Cucumis sativus* L.) seeds coated with *P. chrysogenum* strain Snef1216 significantly increase seed germination and inhibited invasion and development of *M. incognita* as compared to the control. The brinjal or eggplant (*Solanum melongena* L.) seeds treated with *Paecilomyces formosus* strain MD12 efficiently increased their germination and also control *M. incognita* infection [61]. Similarly, tomato seeds coated with *A. tubingensis* considerably enhanced their germination rate in the present investigation and reduced nematode invasion in plant roots.

Tomatoes are susceptible to *M. enetrolobii* infection and serve as a good host for their reproduction [62]. Nematophagous microorganisms are natural predators of nematodes and provide a potentially effective method for managing root-knot nematodes in their natural environment [20]. Previous research showed that *Aspergillus* species can reduce the penetration of nematodes [34,42]. *A. niger* prevented the development of *M. incognita* in tomato [36,47]. *A. tubingensis* has the potential biocontrol or metabolites to inhibit pathogens and control diseases (Kriaa et al., 2015) and enhanced growth [39]. In another study, Zhao et al. [63] reported that *A. tubingensis* strain QF05 effectively (*p* < 0.05) protected tomato plants against grey mould (*Botrytis cinerea*), considerably increased plant length, dry mass, and fresh mass, and successfully colonized the rhizosphere. Our results also revealed that the application of *A. tubingensis* noticeably reduced the population of *M. enterolobii* and root galls in tomato under greenhouse conditions. The application of *A. niger and P. lilacinus* led to a considerable reduction in the soil populations of *M. javanica* and root galling and increased biomass [64]. According to the current study’s findings, it is also clear that the use of *A. tubingensis* considerably decreases the invasion of *M. enetrolobii* and has consequences for increasing the biomass of tomato plants. Moreover, their ability to develop and reproduce was hampered by *A. tubingensis*. Our findings corroborate recent research by Liu et al. [65], which demonstrated that the application of *A. welwitschiae* considerably minimizes the attraction of *A. welwitschiae*-treated rice roots to *M. graminicola* and significantly inhibits nematode penetration and development. In addition, we observed that seed-coated plants had greater efficiency in the greenhouse experiments, both in terms of preventing nematode infestation and accelerating the growth of the plants. The present study revealed that *A. tubingensis* GX3 strain can be used as a novel microbial biocontrol agent against *M. enterolobii* in tomato.

## 4. Materials and Methods

### 4.1. The Activation of the Fungus Strain and the Preparation of Fermentation Broth

*A. tubingensis* was collected from Xiaopingyang Town, Xingbin District, Laibin City, Guangxi, China (109°07′45″ E, 23°24′13″ N) from maize fields exhibiting natural declines of *Heterodera zeae* (Koshy, Swarup and Sethi, 1971) (maize cyst). The fungal mycelium was first transferred into the PDA-filled cavities. After that, the isolates were purified and tested for their infectivity on *M. enterolobii*, and the parasitism rate of strain GX3 was 100%. The strain was stored at −80 °C in Guangxi Key Laboratory of Agric-Environment and Agric-Products Safety, Guangxi University, China.

The culture of the fungus *A. tubingensis* GX3 strain (GenBank: OQ830475) was grown in a PDA medium at 25 °C for five to seven days in order to check the potency and activate the strain. A 9 mm disk in diameter was taken from the margin of the single pure fungal colony and placed into a 250-milliliter triangle flask that contained 100 mL of sterile Czapek media (C_12_H_22_O_11_ 3.0 g, NaNO_3_ 0.2 g, K_2_HPO_4_ 0.1 g, MgSO_4_.7H_2_O 0.05 g, KCl 0.05 g, FeSO_4_.7H_2_O 0.001 g, H_2_O 100 mL). After that, the flasks were agitated at 160 rpm in an MQD-S2R shaker (manufactured by Minquan Instrument Co., Ltd., in Shanghai, China) for a maximum of four weeks at a temperature of 25 °C. After four weeks, the fermentation broth was filtered with 0.22-micron Millipore filters (Whatman, Clifton, NJ, USA). The fermentation was kept refrigerated at 4 °C in order to be used later [44].

### 4.2. Nematode Culture

The root-knot nematode *M. enterolobii* was cultured on tomato roots from a single egg mass in the nematology laboratory at Guangxi University in Nanning, China. After forty days, infected plants were removed, cleansed, and cut into 3–4 cm pieces. Sodium hypochlorite (0.05%) was added and agitated for 1–2 min to break down the egg-mass matrix. The mixture was sieved through 500 mesh and rinsed with distilled water to remove sodium hypochlorite. About 10 mL of 454 g L^−1^ of sucrose was added, and the eggs were centrifuged at 3000 rpm for four minutes to further clean the eggs. Then, the supernatant was poured into a 500-sieve mesh and washed with sterile distilled water. Using the modified Baermann funnel procedure, the eggs were allowed to hatch in sterilized distilled water in the darkness at 28 °C and 85% humidity level [60].

### 4.3. Effect of Fermentation Filtrate on M. enterolobii Juveniles (J2s)

About one hundred freshly hatched J2s were placed in each well of the 96-well plate, together with 200µL of fermentation filtrate at different concentrations (100%, 75%, 50%, 25%, 10%, and 5%), Czapek medium, and distilled water. The microliter plate was placed in an incubator at 28 °C under humidified conditions for 72 h. At 6, 12, 24, 48, and 72 h, the number of dead nematodes was counted using a microscope (ECLIPSE, Ti-S, or Nikon, Tokyo, Japan). Their morphological pictures were taken after 72 h with the microscope (Axio Imager, Z2, Carl Zeiss, Jena, Germany). To ascertain whether the fermentation of *A. tubingensis* had larvistatic or larvicidal ability, the immotile J2s were rinsed and transferred into distilled water to observe a restoration of motility. Nematodes were considered dead when straight, stiff, and immotile after transfer to sterilized tap water for 12 h, even when agitated with a fine hair needle [66]. The experiment was repeated three times, with each treatment done in triplicate. Using the following formula, we were able to calculate the mortality rate of J2s:

Mortality % = (number of dead juveniles)/(total number of juveniles) × 100(1)

### 4.4. Effect of Fermentation Filtrate on M. enterolobii Egg Hatching

About one hundred eggs, 200 µL of fermentation filtrate at varied concentrations (100%, 75%, 50%, 25%, 10%, and 5%), Czapek medium, and distilled water were poured into each 96-well plate well (He et al., 2020). The microliter plate was covered and put in a 28 °C incubator with constant humidity. An inverted microscope (ECLIPSE, Ti-S, Nikon) was used to observe the egg hatching at 2, 4, 6, and 8 days. Unhatched eggs were placed in distilled water to confirm *A. tubingensis* fermentation’s ovicidal or ovistatic properties [66]. In order to confirm the accuracy of the results, each treatment was carried out in triplicate. The cumulative hatching rate was determined by applying the following formula:

Hatching % = (number of hatched eggs)/(total number of eggs) × 100(2)

The hatching inhibition percentage was determined by using the following formula:

Hatching inhibition % = (Hatching in control − Hatching in treated)/Hatching in control × 100(3)

### 4.5. Sterilization of Seeds and Seeds Coating

The tomato seeds Zhongza 09 cultivar (susceptible to root-knot nematodes) were firstly disinfected for one minute with 75% ethanol (C_2_H_6_O) and 0.05% Tween 20 (C_58_H_114_O_26_). Then, they were disinfected for one minute with 2.5% sodium hypochlorite (NaClO). After that, 95% ethanol (C_2_H_6_O) was added for two to three minutes. After each stage of the disinfection process, the seeds were washed three times with sterile, distilled water. A well-ventilated and dry atmosphere was used to air-dry the seeds. The next step was to coat the dry seeds with *A. tubingensis* GX3′ fermentation broth in a volume ratio of 1:1 (seeds: fermentation), whereas the control seeds were coated with sterile distilled water. After coating, the seeds were allowed to air-dry. For germination, coated and controlled seeds were placed in different Petri dishes containing two moist filter paper layers. The germination percentage (G%), the germination index (GI), and the germination rate (GR) were computed as described by Sikandar et al. [60];

G % = (Number of germinated seeds)/(Total number of seeds) × 100(4)

GI = N.G.S(1)/D.C(1) + N.G.S(2)/D.C.(2) + N.G.S(f)/D.C(f)(5)

whereas N.G.S(1), N.G.S(2), and N.G.S(f) are the numbers of germinated seeds in 1st, 2nd, and final counts. D.C(1), D.C(2), and D.C(f) stand for days to 1st, 2nd, and final counts.

GR = a + (a + b) + (a + b + c)(a + b + c + m)/n(a + b + c + m)(6)

whereas a, b, c are the number of seedling in the first, second, and third counts; m stands for the number of seedling in the final counts, and n stands for number of counts.

### 4.6. Greenhouse Experiments

The two experiments were conducted in the greenhouse to assess the biocontrol efficacy of *A. tubingensis* against *M. enterolobii*. (1) Fermentation broth experiment: About 2000J2s and 4 mL of 100% fermentation broth were added to each pot after six days of transplanting tomato plants. Plants were in the true two-leaf stage at the time of inoculation. (2) Seed-coated experiment: At the true two-leaf stage, each seed-coated plant was inoculated with 2000J2s in a 1–2 cm deep hollow at the base of the stem.

The treatments were CK+ (control with nematodes inoculum), CK− (control without nematodes inoculum), T+ (100% fermentation broth and nematodes inoculum both); T− (100% fermentation broth only), CS+ (coated seeds plants with nematodes inoculum), CS− (coated seeds plants without nematodes inoculum). All treatments had five replications, and each experiment was repeated three times for the accuracy of results.

#### Growth Index Observation and Nematodes Infection Determination

Growth parameters such as plant height (cm), stem diameter (mm), root length (cm), fresh root weight (g), dry root weight (g), fresh stem weight (g), and dry shoot weight (g) were measured. The Quarrie and Jones equation was used to compute leaf area (LA) [67]. Germinated plants were watered with tap water every two days in a greenhouse at 26–32 °C, 14 h light and 10 h dark period, and 85% relative humidity. Five plants were selected randomly from each treatment at 7, 14, 21, and 28 dpi (days post inoculation) for evaluating the variation among growth parameters.

Tomato root samples were collected at 7, 14, 21, and 28 dpi. The roots of five randomly selected plants from each treatment were carefully collected and cleaned to remove any soil adhering. The roots were stained and examined under a microscope (ECLIPSE, Ti-S, Nikon) to determine the RKN stages. The photographs of nematodes were taken with a microscope (Axio Imager, Z2, Carl Zeiss).

### 4.7. Model Validations

The relationship was utilized to build a model for fermentation concentrations and exposure times and to calculate coefficient of determination (R^2^) values with root mean square error (RMSE). The standard deviation of the calculated errors is denoted by RMSE and *R*^2^. The sum of the prediction errors is the number of values that fall outside of and around the regression lines. The RMSE statistic assessed the model’s accuracy across the various concentrations and exposure times. The Debaeke et al. [68] equation was used to compute the RMSE.

### 4.8. Statistical Analysis

All treatments had five replications, and each experiment was repeated three times. The results of repeated experiments were combined. All recorded data were evaluated using one-way ANOVA to determine the significance of all treatments on tomato (ANOVA). Duncan’s multiple range test was used to determine whether there was a statistically significant difference between the treatments (*p >* 0.05). Various statistical programs were used to administer all statistical operations, including IBM-SPSS statistics 25.0 version software, EPA Probit analysis program (version 1.5) software, and Microsoft Excel 2013 [3]. Sigma Plot 10.0 was used to construct the graphs.

## 5. Conclusions

Based on the findings of this study, it appears that *A. tubingensis* can serve as a nematicide. During in vitro experiments, increasing the fermentation concentration and the exposure time increased the mortality rate and inhibition of egg hatching. It has been determined that *M. enterolobii* has reasonable sensitivity toward *A. tubingensis*. In addition, the seed coated with the fermentation of *A. tubingensis* showed plant growth-promoting characteristics and nematicidal activities with potential utility as biocontrol agents against *M. enterolobii* in tomato under greenhouse conditions. It has commercial potential as a microbial biocontrol agent, but further research into the separation and extraction of nematicidal compounds and their modes of action is required before recommending this fungal fermentation as a commercial nematicide. As a consequence of our research, we now have a better understanding of effectively and sustainably controlling the root-knot nematode disease that affects tomato and other host plants. This study also contributes to the development of safer alternatives to chemical nematicides.

## Figures and Tables

**Table 1:** Effect of *A. tubingensis* fermentation against mortality of *M. enterolobii* juveniles.

Fermentation	Mortality of *M. enterolobii* Juveniles				
	6 h	12 h	24 h	48 h	72 h
100% fermentation	22.6 ± 3.05 ^a^	47.2 ± 5.49 ^a^	78.8 ± 3.49 ^a^	99.0 ± 1.41 ^a^	100.0 ± 0 ^a^
75% fermentation	14.2 ± 2.39 ^b^	35.6 ± 3.91 ^b^	51.6 ± 4.22 ^b^	87.0 ± 3.16 ^b^	100.0 ± 0 ^a^
50% fermentation	10.6 ± 2.41 ^c^	25.4 ± 3.05 ^c^	37.0 ± 2.55 ^c^	59.8 ± 6.76 ^c^	97.8 ± 2.49 ^a^
25% fermentation	6.0 ± 1.58 ^d^	17.2 ± 3.42 ^d^	25.0 ± 3.87 ^d^	32.4 ± 3.44 ^d^	77.4 ± 4.39 ^b^
10% fermentation	2.0 ± 1.00 ^e^	11.2 ± 1.79 ^e^	15.8 ± 3.27 ^e^	19.6 ± 2.07 ^e^	37.2 ± 4.49 ^c^
5% fermentation	0.6 ± 0.55 ^e^	4.8 ± 1.64 ^f^	11.4 ± 3.21 ^f^	14.6 ± 3.78 ^f^	26.8 ± 2.59 ^d^
Cazpek Medium	0 ± 0 ^e^	0 ± 0 ^g^	2.0 ± 1.58 ^g^	3.6 ± 1.34 ^g^	8.8 ± 1.48 ^e^
Distilled water	0 ± 0 ^e^	0 ± 0 ^g^	1.8 ± 1.30 ^g^	4.2 ± 1.30 ^g^	8.0 ± 1.58 ^e^

Data represents mean ± standard deviation. The different letters denoted within the column are statistically different according to Duncan multiple range test (*p* > 0.05).

**Table 2:** Effect of *A. tubingensis* fermentation on *M. enterolobii* eggs hatching.

Fermentation	*M. enterolobii* Eggs Hatching			
	2 d	4 d	6 d	8 d
100% fermentation	0 ± 0 ^d^	0 ± 0 ^f^	0 ± 0 ^e^	0.1 ± 0.22 ^f^
75% fermentation	0 ± 0 ^d^	0 ± 0 ^f^	0.1 ± 0 ^e^	0.2 ± 0.45 ^f^
50% fermentation	0 ± 0 ^d^	0 ± 0 ^f^	0.4 ± 0.55 ^e^	0.6 ± 0.89 ^f^
25% fermentation	0 ± 0 ^d^	2.2 ± 2.49 ^e^	2.8 ± 2.39 ^e^	5.4 ± 4.51 ^e^
10% fermentation	0.6 ± 0.5 ^d^	8.4 ± 1.67 ^d^	10.4 ± 2.07 ^d^	16.0 ± 3.32 ^d^
5% fermentation	3.4 ± 1.67 ^c^	13.2 ± 3.27 ^c^	18.6 ± 3.85 ^c^	26.6 ± 4.04 ^c^
Cazpek Medium	20.6 ± 2.88 ^b^	46.0 ± 4.12 ^b^	68.8 ± 6.94 ^b^	87.2 ± 5.26 ^b^
Distilled water	24.8 ± 3.56 ^a^	51.6 ± 3.78 ^z^	87.2 ± 4.55 ^a^	92.0 ± 2.92 ^a^

Data represents mean ± standard deviation. The different letters denoted within the column are statistically different according to Duncan-multiple range test (*p* > 0.05).

**Table 3:** Toxicity of *A. tubingensis* fermentation against *M. enterolobii* juveniles.

Assay	Time (h)	LC_50_ % (95% CI)	LC_90_ % (95% CI)	Slop ± S.E	x^2^
Larvicidal assay	6	437.76 (322.13–659.92)	4357.15 (2385.87–9866.99)	1.28 ± 0.10	3.73
	12	141.39 (101.32–230.21)	1693.06 (795.48–5626.89)	1.19 ± 0.10	16.56
	24	59.22 (35.81–120.05)	413.67 (173.92–6415.08)	1.52 ± 0.32	91.80
	48	39.71 (22.50–52.34)	84.39 (63.16–172.71)	3.91 ± 0.87	111.04
	72	13.09 (9.61–16.65)	34.89 (27.06–49.76)	3.01 ± 0.33	42.48

Whereas LC_50_ (50% Lethal Concentration); LC_90_ (90% Lethal Concentration); CI (Confidence Interval); Slop ± S.E (Slop ± Standard error); x^2^ (Chi-square).

**Table 4:** Median effective concentration of *A. tubingensis* fermentation on *M. enterolobii* eggs hatching.

Assay	Time (d)	EC_50_ % (95% CI)	EC_90_ % (95% CI)	Slop ± S.E	x^2^
Ovicidal assay	2	2.36 (1.40–3.16)	5.97 (4.84–7.32)	3.19 ± 0.54	0.12
	4	2.92 (2.28–3.56)	12.05 (10.42–14.05)	2.08 ± 0.15	10.90
	6	2.32 (2.03–2.62)	10.40 (9.33–11.65)	1.97 ± 0.09	9.19
	8	3.55 (2.34–4.82)	13.74 (10.23–20.26)	2.18 ± 0.23	32.81

Whereas EC_50_ (50% Effective Concentration); EC_90_ (50% Effective Concentration); CI (Confidence Interval); Slop ± S.E (Slop ± Standard error); x^2^ (Chi-square).

**Table 5:** Regression model various concentrations of *A. tubingensis* fermentation in response to larvicidal and ovicidal assay.

Fermentation	Larvicidal			Ovicidal		
	*R* ^2^	RMSE	Regression Eq.	*R* ^2^	RMSE	Regression Eq.
100%	0.93	7.944	y = 20.66x + 7.54	0.60	0.024	y = 0.03x − 0.05
75%	0.98	4.307	y = 22.3x − 9.22	0.60	0.049	y = 0.06x − 0.1
50%	0.94	7.381	y = 20.88x − 16.52	0.90	0.075	y = 0.22x − 0.3
25%	0.83	10.08	y = 15.8x − 15.8	0.95	0.371	y = 1.68x − 1.6
10%	0.92	3.280	y = 7.88x − 6.48	0.95	1.061	y = 4.82x − 3.2
5%	0.95	2.021	y = 6.22x − 7.02	0.99	0.807	y = 7.5x − 3.3
Cazpek medium	0.85	1.264	y = 2.12x − 3.48	0.99	1.576	y = 22.26x − 5 × 10^−14^
Distilled water	0.89	0.988	y = 2.02x − 3.26	0.93	6.315	y = 23.72x + 4.6

**Table 6:** Regression model for exposure time in response to ovicidal and larvicidal assay.

Assay	Time	*R^2^*	RMSE	Regression Equation
Larvicidal assay	6 h	0.87	2.756	y = −3.1333x + 21.1
	12 h	0.95	3.695	y = −6.8595x + 48.543
	24 h	0.90	7.729	y = −10.393x + 74.693
	48 h	0.92	10.11	y = −14.631x + 105.86
	72 h	0.92	10.65	y = −16.11x + 129.49
Ovicidal assay	2 d	0.66	5.642	y = 3.4214x − 9.2214
	4 d	0.76	9.815	y = 7.5833x − 18.95
	6 d	0.73	16.66	y = 12.102x − 30.936
	8 d	0.77	16.16	y = 13.9x − 34.05

## Data Availability

The authors confirm that the data supporting the findings of this study are available within the article.
